# Immunometabolic Reprogramming in Response to HIV Infection Is Not Fully Normalized by Suppressive Antiretroviral Therapy

**DOI:** 10.3390/v14061313

**Published:** 2022-06-15

**Authors:** Pragney Deme, Leah H. Rubin, Danyang Yu, Yanxun Xu, Gertrude Nakigozi, Noeline Nakasujja, Aggrey Anok, Alice Kisakye, Thomas C. Quinn, Steven J. Reynolds, Richard Mayanja, James Batte, Maria J. Wawer, Ned C. Sacktor, Deanna Saylor, Norman J. Haughey

**Affiliations:** 1 Department of Neurology, Johns Hopkins University School of Medicine, Baltimore, MD 21287, USA; pdeme1@jhmi.edu (P.D.); lrubin@jhu.edu (L.H.R.); sacktor@jhmi.edu (N.C.S.); 2 Department of Epidemiology, Johns Hopkins University Bloomberg School of Public Health, Baltimore, MD 21205, USA; tquinn2@jhmi.edu (T.C.Q.); sjr@jhmi.edu (S.J.R.); mwawer1@jhu.edu (M.J.W.); 3 Department of Psychiatry and Behavioral Sciences, Johns Hopkins University School of Medicine, Baltimore, MD 21287, USA; 4 Department of Applied Mathematics and Statistics, Johns Hopkins University, Baltimore, MD 21218, USA; dyu33@jhu.edu (D.Y.); yanxun.xu@jhu.edu (Y.X.); 5 Division of Biostatistics and Bioinformatics at The Sidney Kimmel Comprehensive Cancer Center, Johns Hopkins University School of Medicine, Baltimore, MD 21231, USA; 6 Rakai Health Sciences Program, Kalisizo P.O. Box 279, Uganda; gnakigozi@rhsp.org (G.N.); aanok@rhsp.org (A.A.); akisakye@rhsp.org (A.K.); rmayanja@rhsp.org (R.M.); jbatte@rhsp.org (J.B.); 7 Department of Psychiatry, School of Medicine, Makerere University College of Health Sciences, Kampala P.O. Box 7072, Uganda; drnoeline@yahoo.com; 8 Division of Intramural Research, National Institute of Allergy and Infectious Diseases, National Institutes of Health, Bethesda, MD 20892, USA; 9 Department of Internal Medicine, University Teaching Hospital, Lusaka P.O. Box 50110, Zambia

**Keywords:** HIV infection, antiretroviral therapy, immunometabolism, glucose oxidation, fatty acid oxidation, amino acid catabolism, comorbid conditions

## Abstract

Background: HIV infection results in immunometabolic reprogramming. While we are beginning to understand how this metabolic reprogramming regulates the immune response to HIV infection, we do not currently understand the impact of ART on immunometabolism in people with HIV (PWH). Methods: Serum obtained from HIV-infected (*n* = 278) and geographically matched HIV seronegative control subjects (*n* = 300) from Rakai Uganda were used in this study. Serum was obtained before and ~2 years following the initiation of ART from HIV-infected individuals. We conducted metabolomics profiling of the serum and focused our analysis on metabolic substrates and pathways assocaited with immunometabolism. Results: HIV infection was associated with metabolic adaptations that implicated hyperactive glycolysis, enhanced formation of lactate, increased activity of the pentose phosphate pathway (PPP), decreased β-oxidation of long-chain fatty acids, increased utilization of medium-chain fatty acids, and enhanced amino acid catabolism. Following ART, serum levels of ketone bodies, carnitine, and amino acid metabolism were normalized, however glycolysis, PPP, lactate production, and β-oxidation of long-chain fatty acids remained abnormal. Conclusion: Our findings suggest that HIV infection is associated with an increased immunometabolic demand that is satisfied through the utilization of alternative energetic substrates, including fatty acids and amino acids. ART alone was insufficient to completely restore this metabolic reprogramming to HIV infection, suggesting that a sustained impairment of immunometabolism may contribute to chronic immune activation and comorbid conditions in virally suppressed PWH.

## 1. Author Summary

The continued development of antiretroviral (ARV) drugs has transformed HIV infection from an almost universally fatal infection to a chronic manageable disease. However, people with HIV (PWH) on ART exhibit increased rates of multiple comorbid conditions, compared with uninfected individuals that are thought to be driven by chronic immune activation and the associated inflammatory response. Interventions to modify the immune and/or inflammatory response in PWH have consistently failed. Immune activation is associated with shifts in metabolic preference that require mobilization of key substrates for the preferred pathway. While there is some data identifying mechanisms for immunometabolic reprogramming in the setting of HIV infection, there is no data on how ART impacts metabolic reprogramming in PWH. In this study, HIV-1 infection is associated with increased glucose utilization, the upregulation of the PPP, increased lactate production, impairments in fatty acid β-oxidation, and upregulation of amino acid oxidative catabolism. Following ART, serum levels of ketone bodies, carnitine, and amino acid metabolism were normalized, however glycolysis, PPP, lactate production, and β-oxidation of long-chain fatty acids remained abnormal despite viral suppression. These findings elucidate energetic pathways that remain perturbed in PWH despite ART and identify several possible intervention points for immunomodulation.

## 2. Introduction

Infection by the human immunodeficiency virus (HIV) results in a highly synchronized activation of host cell metabolism that serves to regulate adaptive and innate immune responses. This immunometabolism is regulated primarily by five metabolic pathways that include glycolysis, the pentose phosphate pathway (PPP), the tricarboxylic acid (TCA) cycle, fatty acid metabolism, and amino acid metabolism [1,2]. Immune activation is associated with shifts in metabolic preference that require mobilization of key substrates for the preferred pathway. For example, quiescent T cells primary rely on oxidative phosphorylation in mitochondria for their energetic needs using fatty acids, amino acids, and/or pyruvate [3]. With antigen stimulation and differentiation into effector T cells there is an increased surface expression of glucose and amino acid transporters to provide substrate for shift in metabolic preference to glutaminolysis and aerobic glycolysis [4]. HIV infection of T cells results in a similar shift in metabolic preference with increased glucose and glutamine concentrations and increased glycolysis and glutaminolysis [5,6,7]. Although macrophages have a greater degree of metabolic plasticity compared with T cells, which is associated with their diverse phenotypes, HIV infection of macrophages is likewise associated with increased glutamine metabolism [8,9]. These data demonstrate that immune activation involves a shift in metabolism that servers to regulate immune cell proliferation and function in response to immune challenges, including HIV infection.

In addition to regulating immunometabolism, HIV modifies host cell metabolism to facilitate its replication. Glycolysis and glutaminolysis are the primary pathways mobilized to support HIV replication in addition to fatty acid synthesis, which is important for formation of the viral envelope, and the production of new virions [10]. The blockade of glucose or glutamine metabolism with the glucose analog 2-deoxy-d-glucose or the glutamine analog 6-diazo-5-oxy-_l_-norleucine strongly inhibits HIV replication [5,6,11,12]. Glucose is phosphorylated to glucose 6-phosphate that can enter into either glycolysis or the PPP. The PPP plays a key role in the synthesis of dNTPs that are crucial regulators of HIV reverse transcription [11]. Consistent with a critical role for dNTPs in HIV replication, the cellular restriction factor SAMDH1 (a dNTP triphosphohydrolase) blocks HIV transcription by reducing the dNTP pool [12]. Pyruvate also plays a critical role in HIV replication. Blocking the pyruvate transporter in mitochondria limits the production of acetyl-CoA that feeds into the TCA cycle and blocks HIV replication. In contrast, the inhibition of lactate dehydrogenase that regulates anerobic glycolysis by catalyzing the conversion of pyruvate into lactate promotes HIV replication [5]. Interestingly, HIV latency is associated with a downregulation in glycolysis [13,14,15]. These findings suggest that oxidative phosphorylation and the PPP play important roles in HIV biogenesis.

Infection by HIV is a chronic condition. As such, shifts in immunometabolism that are intended to activate the immune system for the purpose of clearing the infection become chronically modified. People with HIV exhibit several chronic metabolic adaptations that are only partially normalized by antiretroviral therapy (ART), including imbalances in phospholipid, sphingolipid and sterol metabolism [16,17,18], elevations of free fatty acids [19], elevated bile acids [17,20], elevated lactate [21], accumulations of metabolic waste products [22], dysregulations in the metabolism of biogenic amines and amino acids, impaired redox balance [16,23], and chronic inflammation that is in part due to microbial translocation [17]. In addition to these metabolic adaptations to chronic HIV infection, some ART regimens can themselves contribute to a metabolic syndrome that is associated with aberrant immunity [15]. For example, protease inhibitors (PIs), nucleoside reverse transcriptase (NRTI), non-nucleoside reverse transcriptase (NNRTI), fusion (FI), and integrase strand transfer inhibitors (INSTIs) have each been associated with lipodystrophy, hyperlipidemia, and insulin resistance.

Serum levels of energetic substrates reflects whole-body energy metabolism and the bioenergetic cross talk, which occurs between various cell types and organs in order to maintain tissue homeostasis through the regulation of nutrients absorption, uptake, storage, and utilization [24]. While a number of studies have examined discrete aspects of metabolism that are modified by HIV infection, there has not been a comprehensive evaluation of adaptations in immunometabolism that occur with HIV infection, or how this metabolic reprogramming responds to ART. In this study, we evaluate serum bioenergetic adaptations to HIV infection in a cohort of ART-naïve PWH in the Rakai District of Uganda, and the impact of ART in this population on immunometabolic reprogramming.

## 3. Materials and Methods

### 3.1. Human Subjects and Approvals

Participants from the Rakai District of Uganda were enrolled and followed between 2013 and 2017 for a study designed to assess the impact of HIV subtype and ART on cognitive function [25]. This initial study targeted subjects with advanced immunosuppression defined as CD4 count ≤200 cells/μL (*n* = 116; 42%), or moderate immunosuppression defined as CD4 count 350–500 cells/μL (*n* = 162; 58%) [25]. Age- and gender-matched HIV seronegative (HIV_SN_) control subjects (*n* = 300) were enrolled from the same geographic region. This study was reviewed and approved by the Research and Ethics Committee of the Uganda Virus Research Institute, the Ugandan National Council for Science and Technology, and the Western institutional review board in the USA. Serum samples and demographic and clinical data collected during this primary study were used in the current study.

### 3.2. Study Design

Study participants were ≥20 years old with an average age of 35.5 ± 8.5 (mean ± SD). Half (51%) of the study population were male. Fasting blood samples were drawn from HIV-infected study participants at baseline (*n* = 278), and ~2 years after the initiation of ART (median of 720 days; range 676–882). Fasting blood from HIV_SN_ subjects (*n* = 300) was obtained at a single visit. The first-line treatment regimen included the combination of an NRTI, either tenofovir (TDF) or zidovudine (AZT), with a PI, lamivudine (3TC), and NNRTIs, either efavirenz (EFV) or nevirapine (NVP). Detailed demographic and clinical characteristics are provided in Table 1.

### 3.3. Chemicals and Solvents

All chemicals used for metabolite extraction and LC-TripleTOF experiments were ultra-pure LC-MS grade. Acetonitrile and methanol were procured from Honeywell Research Chemicals (Muskegon, MI, USA). Formic acid and 1N HCl were obtained from Sigma-Aldrich (St. Louis, MO, USA). Demineralized water (ddH2O) was generated by Millipore Advantage Milli-Q system (EMD Millipore, Billerica, MA, USA). Internal standards including: glucose-D7, glucose-6-phosphate-13C6, octanoic acid-D15, lauric acid-D23, tyrosine-D2, glutamic acid-D3, citric acid-D4, and chenodeoxycholic acid-D5 were purchased from Cambridge Isotopes Ltd. (Tewksbury, MA, USA). 25-hydroxycholesterol-D6 and lauroyl-l-carnitine-D3-(chloride) were obtained from Cayman Chemicals (Ann Arbor, MI, USA). Valine D5, tryptophan D5, and cytosine-D2 were from CDN isotopes (Quebec, Canada). 1-oleoy-2-hydroxy-sn-glycero-3-phosphocholine-D7 was from Avanti Polar Lipids, Inc. (Alabaster, AL, USA), and caffeine-13C2 was from Toronto Research Chemicals (Toronto, Canada). Mass calibration solutions (APCI -Positive and Negative) were purchased from AB Sciex (P/N: 4460131 and 4460134; Concord, CA, USA).

### 3.4. Metabolite Extraction

Serum samples (150 µL) were protein precipitated using 850 µL of 70% ice-cold methanol (0.5% 1N HCl) containing pre-spiked heavy isotope internal standards (15) that included glucose-D7, glucose-6-phosphate-13C6, octanoic acid-D15, lauric acid-D23, chenodeoxycholic acid-D5, glutamic acid-D3, citric acid-D4, Lauroyl-l-carnitine-D3-(chloride), 25-hydroxycholesterol-D6, 1-oleoy-2-hydroxy-sn-glycero-3-phosphocholine-D7, cytosine-D2, tyrosine-D2, valine D5, tryptophan D5, and caffeine-13C2. These internal standards were carefully chosen to represent structurally diverse classes of metabolites, as the structure of a metabolite determines how efficiently it is extracted and ionized [26]. This combination of internal standards allowed us to monitor extraction, track instrument precision, and accurately quantify multiple classes of metabolites.

The samples were vigorously mixed for 5 min using a TissueLyser LT (Qiagen, Switzerland) at 50 Hz, and centrifuged at 13,000 rpm at 4 °C for 20 min. The supernatants containing internal standards and unknown metabolites were collected and evaporated to complete dryness using a vacuum concentrator (Thermo Scientific “Savant SpeedVac” SPD 120P2, Waltham, MA, USA). The residues were then resuspended in 150 µL of 50% ice-cold methanol containing 0.1% formic acid and two additional standards, L-leucenol and sulfinpyrazone (2 ppm each), which were used to track instrument performance and mass accuracy throughout the analyses. Samples were vortexed, centrifuged at 13,000 rpm at 4 °C for 10 min, and supernatants collected for metabolomic profiling.

### 3.5. UFLC-QTOF-HRMS/MS Analysis

Metabolomic profiling was performed using ultrafast liquid chromatography (Shimadzu, Kyoto, Japan) coupled to a TripleTOF 5600 (AB Sciex, Concord, CA, USA) high-resolution mass spectrometer (UFLC-HRMS/MS). The UFLC system consisted of a degasser, a quaternary pump, an autosampler, and a temperature-controlled column compartment. The TripleTOF 5600 mass spectrometer was equipped with DuoSpray Turbo V-dual ESI/APCI source probes. The HRMS/MS used an automated calibrant delivery system that was delivered every 15 sample injections by manufacturer-recommended calibrants to maintain the mass accuracy of the instrument (<5 ppm). Ten microliters of each extracted sample were subjected to UFLC-HRMS/MS analysis for metabolomic profiling. Metabolites were chromatographically separated on a Kinetex Pentafluorophenyl (PFP/F5) stationary phase column (Phenomenex, Torrance, CA, USA) using a binary gradient mobile phase program (acetonitrile-eluent A and ddH2O-eluent B; both contained 0.1% FA) with the following parameters: 0–3 min 100% eluent B, 3–13 min, eluent A increased from 0% to 100%, 13–19 min eluent A held at 100%; 19–19.10 eluent B increased to 100% from 0%, and was held for 4 min to allow for column equilibration before the next sample is introduced into the system. The UFLC-separated metabolites were introduced into an electrospray ionization chamber for data acquisition in both positive and negative modes separately over a mass range of 30–900 *m*/*z*. Data were acquired and collected in an information-dependent acquisition (IDA-HRMS/MS) metabolomics approach, which obtained accurate *m*/*z* (mass-to-charge ratio) of both the pre-cursor and fragments of each metabolite. The acquired data were processed using Sciex OS -Q1.5 data analysis software (AB SCIEX, Concord, ON, Canada) integrated with the National Institute Of Standards and Technology NIST (2017) tandem mass spectral library, and the Sciex accurate MS/MS spectral library 2.0 for peak detection, peak alignment, spectral processing, feature identification based on matching pairs of precursor (<5 ppm)-fragment ions (<50 ppm) to the tandem MS databases, and peak quantification.

### 3.6. Targeted Metabolites List

In this study, we focused on bioenergetic metabolites known to be associated with glycolysis, the PPP, the TCA cycle, fatty acid beta-oxidation, and ketogenic and glucogenic amino acid metabolism. To identify and generate a reliable and reproducible targeted list of bioenergetic metabolites, we first extracted a pooled serum sample consisting of small aliquots from all participants to identify the features that were highly reproducible. Metabolite extracted from pooled samples were analyzed in eight sequential injections using our LC-HRMS/MS system, as described above. To be included in the targeted list, a given metabolite must be present in 7 of 8 analytical runs with a coefficient of variation (CV) less than 20%. The metabolites meeting these criteria were used to create a targeted list to identify pre-validated metabolites present in experimental samples.

### 3.7. Data Processing and Normalization

To ensure the instrument operated at peak performance over the period required to run 856 samples, we performed source cleaning twice in a day (before submitting morning and evening sample batches). In addition, we performed extensive cleaning of ion path lenses, including Q-JET and QO, after every 50 sample injections. To avoid potential issues with the normalization of metabolite data, we used twelve internal standards that represented each class and subclass of metabolites as described above. Samples in which the extraction solvent internal standards varied above a 30% CV and resuspension solvent internal standards that varied above a 20% CV were re-run.

### 3.8. Statistical Analysis

Brown–Forsythe and Welch ANOVA tests for multiple group comparisons in combination with Games-Howell multiple comparisons correction for a sample size >50 with a 95% CI using GraphPad Prism (La Jolla, CA, USA) software 9.1.6 were performed. All data were expressed as mean with 95% confidence intervals (CIs). Adjusted (95%, CI) *p*-values of less than 0.05 were considered as statistically significant. Correlation analyses between bioenergetic metabolites, clinical and demographics profiles, including age, gender, CD4+ T-cell count, vial load, and liver enzymes; alanine aminotransferase (ALT); and aspartate aminotransferase (AST) were calculated using Pearson’s correlations in the R package. Correlation values were visualized as heat maps using GraphPad Prism (La Jolla, CA, USA) software 9.1.6. Positive correlations were displayed in blue and negative correlations in red. The shade was proportional to the correlation coefficient R-values. Correlational coefficient R-values greater than 0.5 or less than −0.5 with *p*-values < 0.05 were consider as strong associations. The power analysis was performed using MetaboAnalyst 5.0 [27] to identify the effect size of the current metabolomics data. The current samples’ size per group PWH before ART (*n* = 278) and after ART (*n* = 278), and HIV_SN_ (*n* = 300) showed 100% prediction power at an FDR *p*-value less than 0.05. Significance notations of adjusted *p*-values (95% CI) at * = *p* < 0.05, ** = *p* < 0.01, *** = *p* < 0.001, and **** = *p* < 0.0001.

## 4. Results

### 4.1. Clinical and Demographic Characteristics

At baseline, a higher percentage of ART-naïve PWH (*n* = 234; 78%) presented with elevated levels of HDL compared with HIV_SN_ (*n* = 123; 41%) (*p* < 0.001), but no group differences were apparent in LDL. A greater number of HIV_SN_ participants (*n* = 50; 16.7%) were hypertensive compared to ART-naïve PWH (*n* = 25; 9%) (*p* < 0.001), and higher rates of obesity were apparent among HIV_SN_ participants (*n*= 17; 6%) (*p* = 0.03) compared with ART-naïve PWH (*n* = 9; 3%). The majority of PWH were treated with tenofovir/lamivudine/efavirenz (TDF/3TC/EFV, *n* = 256; 92%), and a small number of PWH were treated with tenofovir/lamivudine/nevirapine (TDF/3TC/NVP, *n* = 10; 4%), zidovudine/lamivudine/nevirapine (AZT/3TC/NVP, *n* = 9; 3%), or zidovudine/lamivudine/efavirenz (AZT/3TC/EFV, *n* = 3; 1%) at their baseline visit. Following ART (treatment for median of 720 days; range 676–882 days), PWH had lower CD4 counts [pre-ART: median (IQR), 363.5 (294.2) compared with post-ART: 402.0(258.5), (*p* < 0.001)], higher fasting glucose (*p* < 0.001), higher total cholesterol (*p* < 0.001), and higher HDL/LDL ratios (*p* < 0.001). ART reduced plasma viral loads to below detectable limits in the majority of PWH (*n* = 237, 85.3%), and reduced viral loads in those who did not achieve undetectable plasma HIV from 4.58 (1.2) pre-ART to 2.33 (1.38) post-ART (*n* = 41, 14.7%) [log10 copies median (IQR)]. Despite ART, 31 (11.1%) PWH still showed advanced immunosuppression (CD4 count ≤200 cells/μL), and 78 (28.1%) had moderate immunosuppression (CD4 count 350–500 cells/μL) at their follow-up visit (Table 1).

### 4.2. Glycolysis, Pentose Phosphate Pathway, and Lactate Metabolism

**Figure 1:**
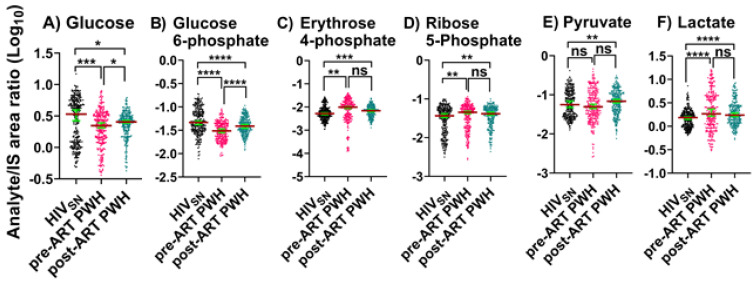

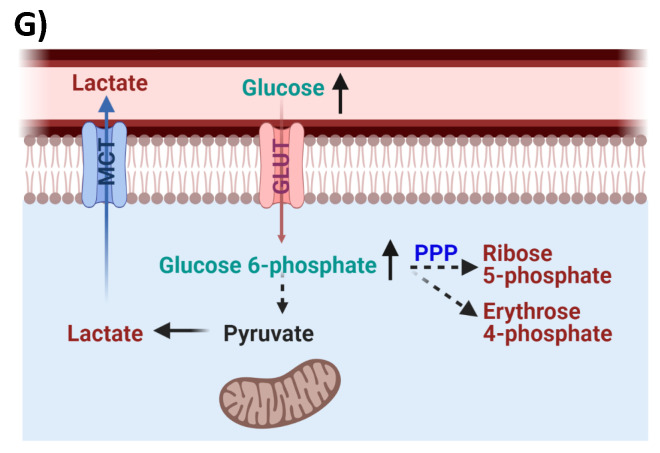
**Glycolysis, PPP, and Lactate metabolism.** Serum levels of (**A**) glucose, (**B**) glucose 6-phosphate, (**C**,**D**) PPP products, (**E**) pyruvate, and (**F**) lactate in PWH before and after ART compared with HIV_SN_. Scatter plots show mean and significance notations of adjusted *p*-values (95% CI) at * = *p* < 0.05, ** = *p* < 0.01, *** = *p* < 0.001, and **** = *p* < 0.0001. For multiple group comparisons, Brown–Forsythe and Welch ANOVA tests were performed in combination with Games-Howell multiple comparisons correction for a sample size >50 with a 95% confidence interval. (**G**) Diagrammatic representation of metabolic pathways modified by HIV and ART. In pre-ART HIV blue text indicates pathway name, black text indicates no change, red text indicates significantly increased levels, and aqua text indicates significantly decreased levels compared with HIV_SN._ Solid line up-arrows (↑) indicate significantly increased and solid line down-arrows (↓) indicate significantly decreased levels following ART, compared with pre-ART HIV.

*Pre-ART PWH compared with HIV_SN_*: Serum levels of glucose (*p* = 0.001) and glucose 6-phosphate (G-6P) (*p* < 0.001) were lower in pre-ART PWH compared with HIV_SN_ (Figure 1A,B). Serum lactate (*p* < 0.001) (Figure 1F) and PPP metabolites, including ribose 5-phosphate (R-5P) (*p* = 0.009), and erythrose 4-phosphate (E-4P) (*p* = 0.004) were higher in pre-ART PWH compared with HIV_SN_ (Figure 1C,D). Pyruvate (*p* = 0.19) levels were similar between pre-ART PWH and HIV_SN_ (Figure 1E).

*Post-ART PWH compared to pre-ART*: ART partially lowered the serum levels of R-5P (*p* = 0.96), E-4P (*p* = 0.7), and lactate (*p* = 0.2), and partially elevated glucose (*p* = 0.02) and G-6P (*p* < 0.001) compared to pre-ART PWH.

*Post-ART PWH compared with HIV_SN_*: Serum levels of glucose (*p* = 0.01) and G-6P (*p* < 0.001) remained low, while R-5P (*p* = 0.002), E-4P (*p* < 0.001), and lactate (*p* < 0.001) remained elevated in post-ART PWH compared with HIV_SN_ (Figure 1A–D,F). Compared to HIV_SN_, pyruvate levels were not changed by HIV infection (*p* = 0.38), but were elevated following ART (*p* = 0.01) (Figure 1E).

These results indicate that ART does not completely restore metabolic dysregulations linked to glycolysis, PPP metabolism, and lactate production in PWH, and suggest that ART may itself dysregulate pyruvate metabolism.

### 4.3. TCA Cycle

We identified five TCA cycle metabolites in serum that included citrate, aconitate, α-ketoglutarate (α-KG), succinate, and malate.

**Figure 2:**
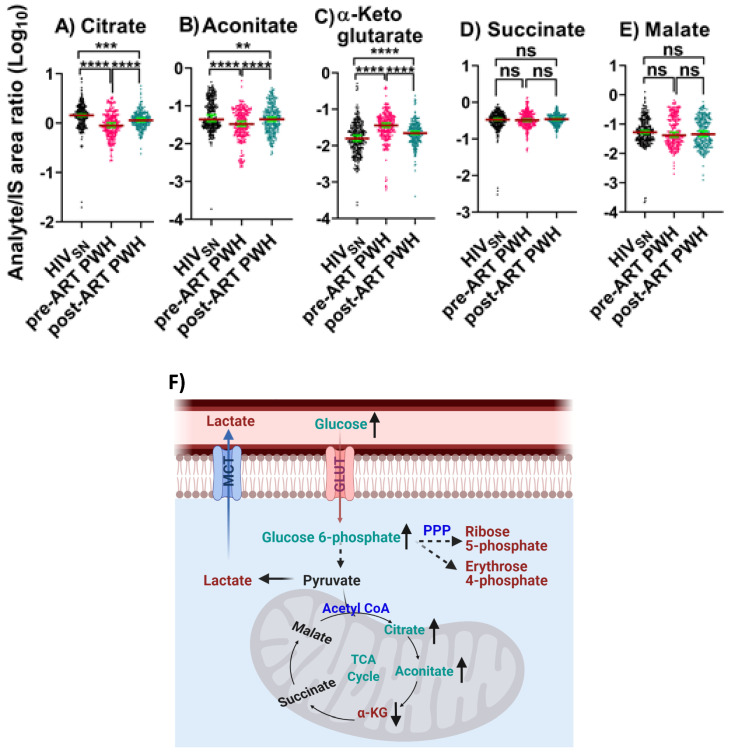
**TCA cycle intermediates**. The levels of (**A**) citrate, (**B**) aconitate, (**C**) α-KG, (**D**) succinate, and (**E**) malate in PWH before and after ART compared with HIV_SN_. Scatter plots show mean and significance notations of adjusted *p*-values (95% CI) at ** = *p* < 0.01, *** = *p* < 0.001, and **** = *p* < 0.0001. For multiple group comparisons, Brown–Forsythe and Welch ANOVA tests were performed in combination with Games-Howell multiple comparisons correction for sample sizes >50 with a 95% confidence interval. (**F**) **Diagrammatic representation of metabolic pathways modified by HIV and ART**. In pre-ART HIV blue text indicates pathway name, black text indicates no change, red text indicates significantly increased levels, and aqua text indicates significantly decreased levels compared with HIV_SN._ Solid line up-arrows (↑) indicate significantly increased and solid line down-arrows (↓) indicate significantly decreased levels following ART compared with pre-ART HIV.

***Pre-ART PWH compared with HIV_SN_*:** In pre-ART PWH, the serum levels of citrate (*p* < 0.001) and aconitate (*p* < 0.001) were lower, while α-KG levels were higher (*p* < 0.001) compared with HIV_SN_ (Figure 2A–C). Succinate (*p* = 0.4) and malate levels (*p* = 0.9) were similar in pre-ART PWH compared with HIV_SN_ (Figure 2D–E).

*Post-ART PWH compared to pre-ART*: ART partially elevated the serum levels of citrate (*p* < 0.001) and aconitate (*p* < 0.001), and partially reduced α-KG (*p* < 0.001) compared to pre-ART PWH.

*Post-ART PWH compared with HIV_SN_*: Citrate (*p* < 0.001) and aconitate (*p* = 0.006) remained low and α-KG (*p* = 0.00006) remained high in post-ART PWH, compared with HIV_SN_ (Figure 2A–C).

These results suggest that ART only partially normalized TCA cycle dysregulation in PWH.

### 4.4. Fatty Acid Metabolism and β-Oxidation

**Figure 3:**
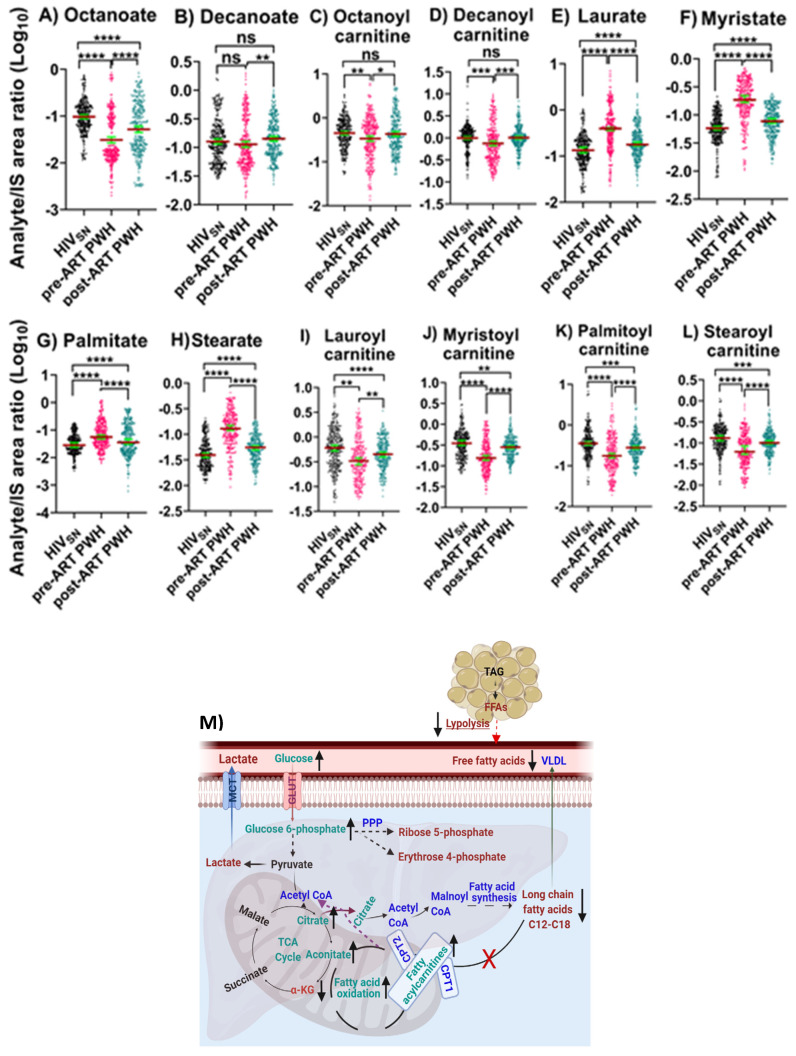
**Fatty acid β-oxidation, fatty acids, and their carnitines**. The levels of (**A**) octanoate, (**B**) decanoate, (**C**) octanoylcarnitine, (**D**) decanoylcarnitine, (**E**) laurate, (**F**) myristate, (**G**) palmitate, (**H**) stearate, (**I**) lauroylcarnitine, (**J**) myristoylcarnitine, (**K**) palmitoylcarnitine, and (**L**) stearoylcarnitine in PWH before and after ART compared with HIV_SN_. Scatter plots show the mean and significance notations of adjusted *p*-values (95% CI) at * = *p* < 0.05, ** = *p* < 0.01, *** = *p* < 0.001, and **** = *p* < 0.0001. For multiple group comparisons, Brown–Forsythe and Welch ANOVA tests were performed in combination with Games-Howell multiple comparisons correction for sample sizes >50 with a 95% confidence interval. (**M**) **Diagrammatic representation of metabolic pathways modified by HIV and ART**. In pre-ART HIV blue text indicates CoA conjugate, enzyme, and pathway names; black text indicates no change; red text indicates significantly increased levels; and aqua text indicates significantly decreased levels compared with HIV_SN_. Solid line up-arrows (↑) indicate significantly increased and solid line down-arrows (↓) indicate significantly decreased levels following ART compared with pre-ART HIV.

*Pre-ART PWH compared with HIV_SN_*: In pre-ART PWH, serum octanoic acid C8 (*p* < 0.001) was low, decanoic acid C10 (*p* = 0.32) levels were unchanged (Figure 3A,B), and their corresponding fatty acylcarnitine (FAC), including octanoylcarnitine (*p* = 0.001) and decanoylcarnitine (*p* < 0.001), were lower compared with HIV_SN_ (Figure 3C,D). Serum long-chain saturated FFAs (LCSFFAs), including laurate (*p* < 0.001), myristate (*p* < 0.001), palmitate (*p* < 0.001), and stearate (*p* < 0.001), were higher (Figure 3E–H), while their corresponding FACs, including lauroyl (*p* < 0.001), myristoyl (*p* < 0.001), palmitoyl (*p* < 0.001), and stearoyl (*p* < 0.001), were lower among pre-ART PWH compared with HIV_SN_ participants (Figure 3I–L).

*Post-ART PWH compared to pre-ART*: ART elevated the levels of octanoate (*p* < 0.001), and decanoate (*p* = 0.01) (Figure 3A,B), and their corresponding octanoylcarnitine (*p* = 0.02) and decanoylcarnitine (*p* < 0.001) (Figure 3C,D). In addition, following ART, serum concentrations of LCSFFAs (Figure 3E–H) were considerably lowered, and their corresponding FACs were vastly elevated compared to pre-ART PWH (Figure 3I–L).

*Post-ART PWH compared with HIV_SN_*: Octanoate partially normalized following ART (but remained low compared with HIV_SN_; *p* < 0.001) (Figure 3A), while octanoylcarnitine and decanoylcarnitine completely normalized compared with HIV_SN_ (Figure 3C,D). Despite ART, serum LCSFFAs remained elevated while their corresponding FACs remained low compared with HIV_SN_ (Figure 3C–F,I–L).

**Figure 4:**
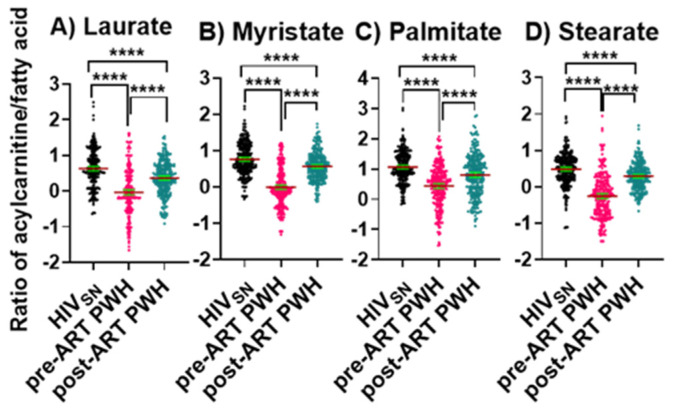
**Long-chain fatty acylcarnitine and their corresponding fatty acid ratios.** The ratios of (**A**) lauroylcarnitine to laurate, (**B**) myristoylcarnitine to myristate, (**C**) palmitoylcarnitine to palmitate, and (**D**) stearoylcarnitine to stearate in PWH before and after ART compared with HIVS_N_. Scatter plots show mean and significance notations of adjusted *p*-values (95% CI) at **** = *p* < 0.0001. For multiple group comparisons, Brown–Forsythe and Welch ANOVA tests were performed in combination with Games-Howell multiple comparisons correction for sample sizes >50 with a 95% confidence interval.

We then calculated the ratios between long-chain FACs to their FFA (C_12_–C_16_) levels in individual subjects to determine the fatty acid β-oxidation status among HIV_SN_, pre-ART, and post-ART PWH. In pre-ART PWH, the serum ratios of the corresponding acyl-carnitine to laurate (*p* < 0.001), myristate (*p* < 0.001), palmitate (*p* < 0.001), and stearate (*p* < 0.001) were low, compared with HIV_SN_ (Figure 4A–D). These ratios significantly increased following ART initiation (Figure 4A–D), but remained low compared with HIV_SN_ (Figure 4A–D). Together, these data suggest that ART partially restored the β-oxidation of LCFFAs, and almost completely restored the metabolism of medium-chain fatty acids.

### 4.5. Amino Acid Metabolism

**Figure 5:**
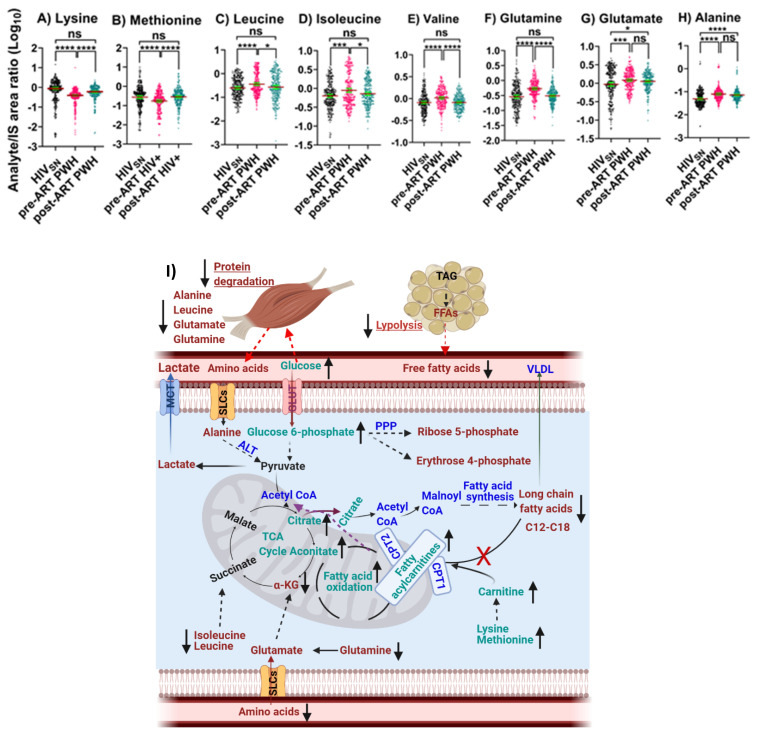
**Amino acid oxidative catabolism.** The levels of (**A**) lysine, (**B**) methionine, (**C**) leucine, (**D**) isoleucine, (**E**) valine, (**F**) glutamate, (**G**) glutamine, and (**H**) alanine in PWH before and after ART compared with HIV_SN_. Scatter plots show mean and significance notations of adjusted *p*-values (95% CI) at * = *p* < 0.05, *** = *p* < 0.001, and **** = *p* < 0.0001. For multiple group comparisons, Brown–Forsythe and Welch ANOVA tests were performed in combination with Games-Howell multiple comparisons correction for sample sizes >50 with a 95% confidence interval. (**I**) **Diagrammatic representation of metabolic pathways modified by HIV and ART**. In pre-ART HIV blue text indicates CoA conjugate, enzyme, and pathway names; black text indicates no change; red text indicates significantly increased levels; aqua text indicates significantly decreased levels, compared with HIV_SN_. Solid line up-arrows (↑) indicate significantly increased and solid line down-arrows (↓) indicate significantly decreased levels following ART compared with pre-ART HIV.

*Pre-ART PWH compared with HIV_SN_*: Serum levels of lysine (*p* < 0.001) and methionine (*p* < 0.001) were low (Figure 5A,B), while leucine (*p* < 0.001), isoleucine (*p* < 0.001), valine (*p* < 0.001), glutamate (*p* < 0.001), glutamine (*p* < 0.001), and alanine (*p* < 0.001) were high (Figure 5C–H) among pre-ART PWH compared with HIV_SN_.

*Post-ART PWH compared to pre-ART*: ART elevated the serum levels of lysine (*p* < 0.001), methionine (*p* < 0.001) (Figure 5A,B) and reduced the levels of leucine (*p* = 0.01), isoleucine (*p* = 0.014), valine (*p* < 0.001), and glutamine (*p* < 0.001) among post-ART PWH to levels not different from HIV_SN_ (Figure 5C–F).

*Post-ART PWH compared with HIV_SN_*: Serum levels of glutamate (*p* = 0.02) and alanine (*p* < 0.001) remained elevated, compared with HIV_SN_ (Figure 5G,H).

### 4.6. Carnitine Metabolism

**Figure 6:**
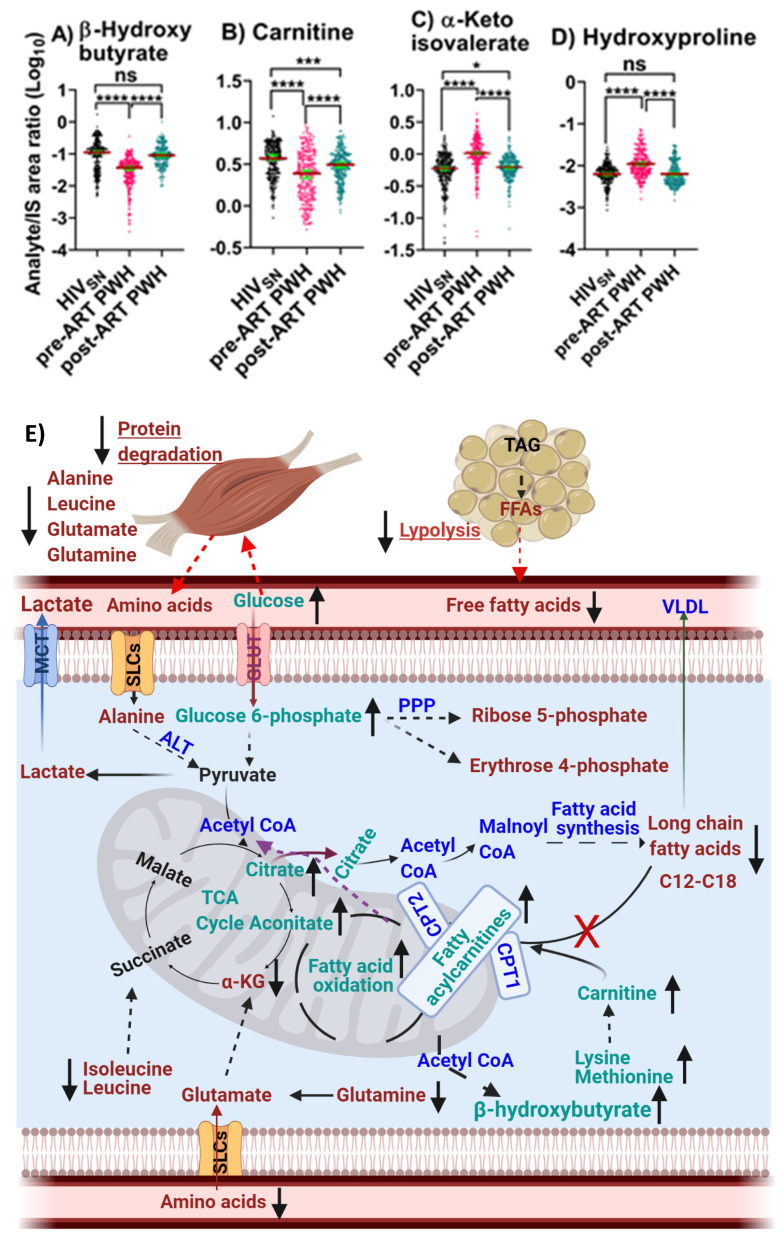
**β-Hydroxybutyrate and amino acid derivative metabolism**. The levels of (**A**) β-hydroxybutyrate, (**B**) carnitine, (**C**) α-ketoisovalerate, and (**D**) hydroxyproline in PWH before and after ART compared with HIV_SN_. Scatter plots show mean and significance notations of adjusted *p*-values at * = *p* < 0.05, *** = *p* < 0.001, and **** = *p* < 0.0001. For multiple group comparisons, Brown–Forsythe and Welch ANOVA tests were performed in combination with Games-Howell multiple comparisons correction for sample sizes >50 with a 95% confidence interval. (**E**) **Diagrammatic representation of metabolic pathways modified by HIV and ART**. In pre-ART HIV, blue indicates CoA conjugate, enzyme, and pathway names; black indicates no change; red indicates significantly increased levels; and aqua indicates significantly decreased levels, compared with HIV_SN_. Solid line up-arrows (↑) indicate significantly increased and solid line down-arrows (↓) indicate significantly decreased levels following ART compared with pre-ART HIV.

*Pre-ART PWH compared with HIV_SN_*: Serum levels of β-hydroxybutyrate (*p* < 0.001) (Figure 6A) and free carnitine (*p* < 0.001) (Figure 6B) were low, while α-ketoisovalerate (α-KIV, *p* < 0.001) (Figure 6C) and hydroxyproline (*p* < 0.001) (Figure 6D) were high among pre-ART PWH compared with HIV_SN._

*Post-ART PWH compared to pre-ART*: β-hydroxybutyrate (*p* < 0.001) (Figure 6A) and free carnitine (*p* < 0.001) (Figure 6B) were elevated, while α-ketoisovalerate (α-KIV, *p* < 0.001) (Figure 6C) and hydroxyproline (*p* < 0.001) (Figure 6D) were reduced following ART compared with pre-ART.

*Post-ART PWH compared with HIV_SN_*: ART completely restored serum β-hydroxybutyrate and hydroxyproline to levels in post-ART PWH to levels not different from HIV_SN_ (Figure 6A,D). Carnitine (*p* < 0.001) and α-KIV (*p* = 0.03) (Figure 6B,C) levels were partially restored among post-ART PWH, compared with HIV_SN_.

Together, the results suggest that ART restored carnitine metabolism as evidenced by improved levels of lysine, methionine, free carnitine, and fatty acyl carnitine. Likewise, ART appears to have improved protein degradation as evidenced by the restoration of branched-chain amino acid levels that included isoleucine, leucine, and valine; decreased levels of α-KIV; a deaminated α-keto acid from valine; and hydroxyproline levels. ART also appears to have partially restored fatty acid β-oxidation, as evidenced by a complete normalization of β-hydroxybutyrate, and partial restoration of circulating LCSFFAs and their corresponding acylcarnitines. The mass spectral data of serum bioenergetics, demographic and clinical data are provided in supplementary materials.

### 4.7. Relationships of Bioenergetic Substrates with Demographic and Clinical Features

**Figure 7:**
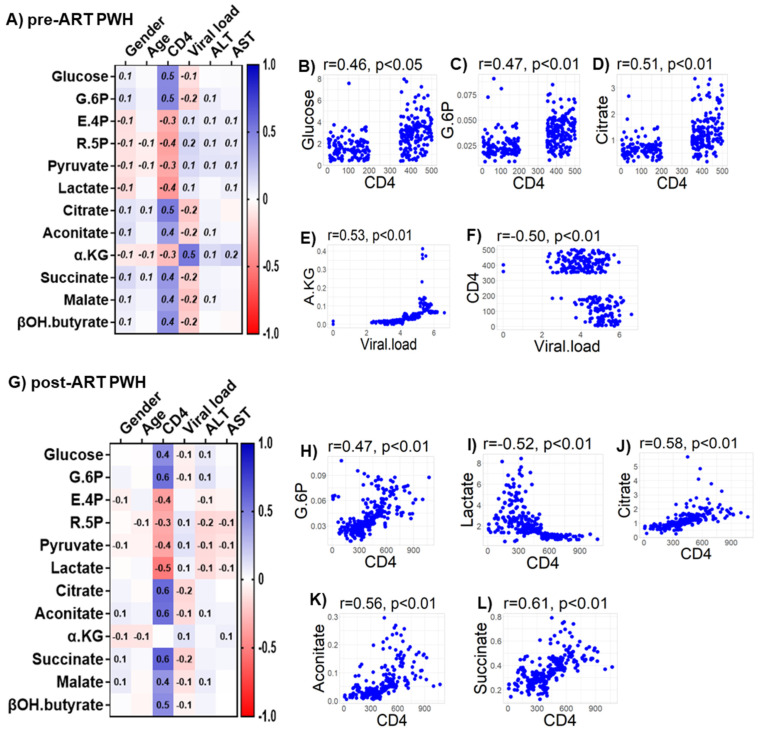
Associations between serum metabolites, demographics, and clinical markers in PWH. Heatmap visualizations of the correlations between bioenergetics substrates and age, gender, biomarkers of liver function (ALT and AST), and biomarkers of HIV disease status (CD4+ T-cell count and HIV viral load) among PWH (**A**) before ART and (**G**) after ART. Numbers in the heatmap cells are Pearson’s correlation coefficient R-values. Blue represents positive correlations and red represents negative correlations. Linear regression graphs between significantly associated covariates. (**B**) Glucose vs. CD4 (0.46), (**C**) G6P vs. CD4 (0.47), (**D**) citrate vs. CD4 (0.51), (**E**) α-KG vs. viral load (0.53), and (**F**) CD4 vs viral load (−0.50) in pre-ART PWH. (**H**) G.6P vs. CD4 (0.47), (**I**) lactate vs. CD4 (−0.52), (**J**) citrate vs. CD4 (0.58), (**K**) aconitate vs. CD4 (0.56), and (**L**) succinate vs. CD4 (0.61) in post-ART PWH.

**Figure 8:**
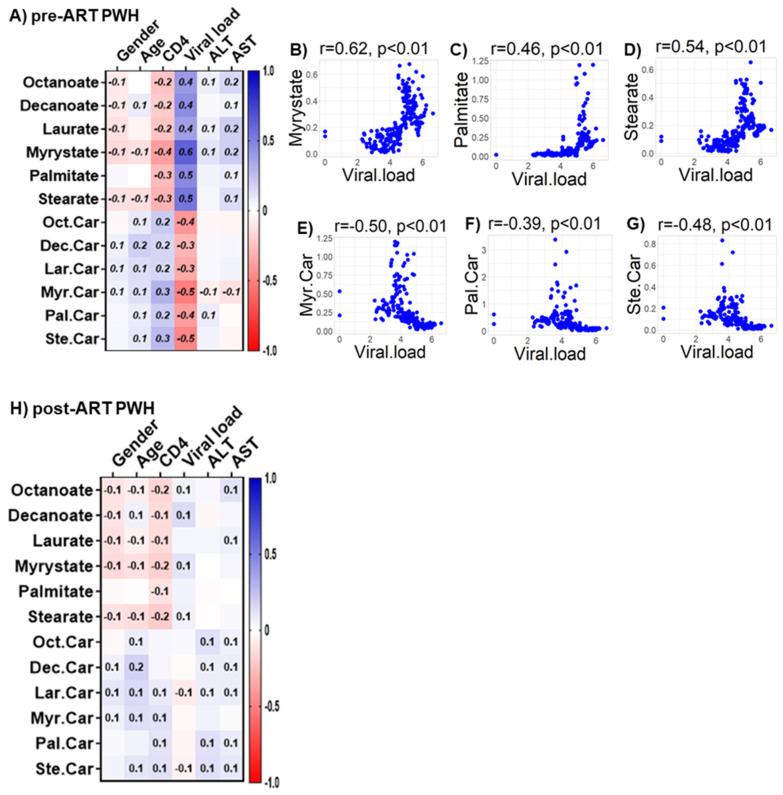
**Associations between serum metabolites, demographics, and clinical markers in PWH.** Heatmap visualizations of the correlations between FAO metabolic substrates and age, gender, biomarkers of liver function (ALT and AST), and biomarkers of HIV disease status (CD4+ T-cell count and HIV viral load) among PWH (**A**) before ART and (**H**) after ART. Numbers in the heatmap cells are Pearson’s correlation coefficient R-values. Blue represents positive correlations and red represents negative correlations. Linear regression graphs between significantly associated covariates. (**B**) myristate vs. viral load (r = 0.62), (**C**) palmitate vs. viral load (r = 0.46), (**D**) stearate vs. viral load (r = 0.54), (**E**) myristoylcarnitine vs. viral load (r = −0.50), (**F**) palmitoylcarnitine (r = −0.39), and (**G**) stearoylcarnitine vs. viral load (r = −0.48) in pre-ART PWH.

**Figure 9:**
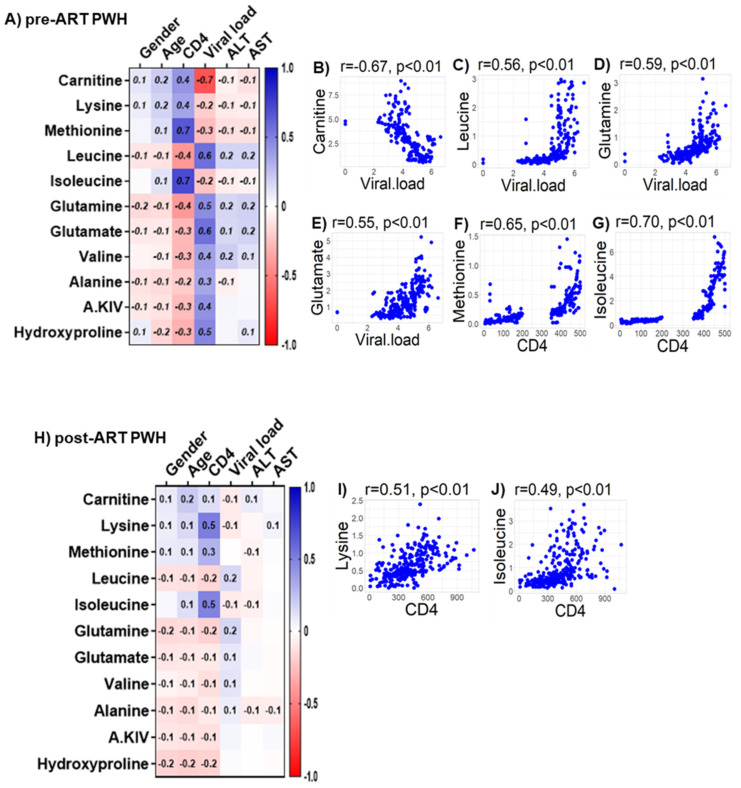
**Associations between serum metabolites, demographics, and clinical markers in PWH.** Heatmap visualizations of the correlations between AA oxidative catabolism substrates and age, gender, biomarkers of liver function (ALT and AST), and biomarkers of HIV disease status (CD4+ T-cell count and HIV viral load) among PWH (**A**) before ART and (**H**) after ART. Numbers in the heatmap cells are Pearson’s correlation coefficient R-values. Blue represents positive correlations and red represents negative correlations. Linear regression graphs between significantly associated covariates. (**B**) Carnitine vs. viral load (r = −0.7), (**C**) leucine vs. viral load (r = 0.56), (**D**) glutamine vs. viral load (r = 0.59), (**E**) glutamate vs. viral load (r = 0.55), (**F**) methionine vs. CD4 (r = 0.65), and (**G**) isoleucine vs. CD4 (r = 0.71) in pre-ART PWH. (**I**) Lysine vs. CD4 (r = 0.51) and (**J**) isoleucine vs. CD4 (r = 0.49) in post-ART PWH.

We observed only weak associations between age and sex or biomarkers of liver function (ALT and AST) with any of the bioenergetic substrates: Glucose oxidation products (Figure S1A); fatty acid oxidation products (Figure S1B); amino acids catabolism products (Figure S1C) in HIV_SN_. In PWH, several of the glucose oxidation metabolites were associated with CD4+ T-cell counts and serum viral loads before (Figure 7A) and after ART (Figure 7G). Higher glucose (r = 0.46), G-6P (r = 0.47) and the TCA cycle product citrate (r = 0.51) were associated with higher CD4+ T-cell counts in pre-ART PWH (Figure 7B–D). Likewise, higher levels of α-KG (r = 0.53) were associated with higher serum viral loads in pre-ART PWH (Figure 7E). Not surprisingly, higher viral loads were associated with lower CD4+ T-cell counts among pre-ART PWH (Figure 7F). The levels of G-6P (r = 0.47), citrate (r = 0.58), aconitate (r = 0.56), and succinate (r = 0.61) were positively correlated, while lactate levels (r = −0.52) were negatively correlated with CD4+ T-cell counts among post-ART PWH (Figure 7H–L). Numerous FFAs and their corresponding FACs were strongly associated with serum viral load in pre-ART PWH (Figure 8A). Metabolites involved in the FAO pathway were not associated with any clinical parameters we assessed in post-ART PWH (Figure 8H). Levels of myristate (r = 0.62), palmitate (r = 0.46), and stearate (r = 0.54) were positively associated, while their acylcarnitines myristoylcarnitine (r = −0.50), palmitoylcarnitine (r = −0.39), and stearoylcarnitine (r = −0.48) were negatively associated with serum viral loads among pre-ART PWH (Figure 8B–G). Serum levels of multiple amino acids were associated with CD4+ T-cell counts and viral loads in PWH before (Figure 9A) and after ART (Figure 9H). These included carnitine (r = −0.67), which was negatively associated with serum viral loads, and leucine (r = 0.56), glutamine (r = 0.59), and glutamate (r = 0.55), which were positively associated with serum viral loads among pre-ART PWH (Figure 9B–E). Higher levels of methionine (r = 0.65) and isoleucine (r = 0.70) were associated with higher CD4+ T-cell counts in pre-ART PWH (Figure 9F–G). Similarly, higher levels of lysine (r = 0.51) and isoleucine (r = 0.49) were associated with higher CD4+ T-cell counts in post-ART PWH (Figure 9I,J).

### 4.8. Metabolic Adaptations Associated with Complete vs. Incomplete Suppression of HIV with ART

We next determined if the degree of metabolic reprogramming in PWH on ART was different when we excluded individuals with detectable viral loads (41 of 278) from the analysis compared to all the subjects. We found a small number of metabolites that were different in abundance when we removed PWH on ART with detectable viral loads from the analysis as described below.

*Post-ART PWH compared with HIV_SN_:* Before the exclusion of post-ART PWH with detectable viral loads, the levels of aconitate were low among post-ART PWH compared with HIV_SN_ (*p* = 0.005). Following exclusion, aconitate levels in post-ART PWH were similar to HIV_SN_ (*p* = 0.07) (Figure S2A). Likewise, levels of succinate that were similar (*p* = 0.1) between post-ART PWH and HIV_SN_ were lower in post-ART PWH when post-ART PWH with detectable viral loads were removed from the analysis (*p* = 0.02) (Figure S2B). Levels of glutamate that were higher among post-ART PWH compared with HIV_SN_ (*p* = 0.009) were similar in post-ART PWH compared with HIV_SN_, when post-ART PWH with detectable viral loads were removed from the analysis (*p* = 0.056) (Figure S2C**)**. Levels of carnitine that were lower in post-ART PWH compared with HIV_SN_ (*p* = 0.0001) were similar to HIV_SN_ after exclusion of post-ART PWH with detectable serum viral loads (*p* = 0.99) (Figure S2D).

*Post-ART compared with pre-ART PWH:* Levels of decanoate that were higher among post-ART PWH compared with pre-ART PWH (*p* = 0.009) were similar when post-ART PWH with detectable viral loads was removed from analysis (*p* = 0.056) (Figure S2E). Likewise, levels of alanine that were similar between post-ART PWH and pre-ART PWH (*p* = 0.18) were lower in post-ART compared with pre-ART PWH (*p* = 0.02), when post-ART PWH with detectable viral loads was removed from analysis (Figure S2F).

### 4.9. Associations of Clinical Measures with Metabolites in PWH on ART with Complete vs. Incomplete Viral Suppression

We determined if the strength of the associations between age, sex, biomarkers of liver function (ALT and AST), biomarkers of HIV disease status (CD4+ T-cell count and HIV viral load), and immunometabolites: Glucose oxidation products (Figure S3A); fatty acid oxidation products (Figure S3B); amino acids catabolism products (Figure S3C) were different among post-ART PWH when we excluded PWH on ART (41 of 278) with detectable viral loads from the analysis. Removing subjects with incomplete viral suppression from the analysis did not robustly modify the results of correlational analyses. The levels of glucose (r = 0.47), G-6P (r = 0.60), citrate (r = 0.56), aconitate (r = 0.58), succinate (r = 0.62), malate (r = 0.4), βOH butyrate (r = 0.4) (Figure S3A), lysine (r = 0.51), methionine (r = 0.3), and isoleucine (r = 0.52) (Figure S3C) were positively correlated, while E4P (r = −0.3), R5P (r = −0.3) and lactate levels (r = −0.6) negatively correlated with CD4+ T-cell counts (Figure S3A) among post-ART PWH with undetectable viral loads.

## 5. Discussion

Our results suggest that HIV-1 infection is associated with several prominent changes in immunometabolism that include: (1) enhanced glucose demand, (2) upregulation of PPP that likely directs glucose utilization to biomolecular synthesis instead of ATP production, (3) increased lactate production that limits pyruvate utilization in the TCA cycle via oxidative phosphorylation in mitochondria, (4) impairment in the β-oxidation of fatty acids, and (5) upregulation in the oxidative catabolism of amino acids. After approximately 2 years of ART, long-chain fatty acid oxidation and PPP remained impaired, and a number of other bioenergetic pathways were not fully restored.

### 5.1. Glucose Metabolism and the Pentose Phosphate Pathway

Previous studies demonstrate that HIV infection is associated with an increased rate of resting energy expenditure [28,29]. Lower circulating glucose levels have been reported in ART-naïve women with HIV compared with uninfected individuals from Rwanda (Africa) [30], and in PWH receiving ART from an Indian cohort [23]. These results are consistent with a higher energy demand in PWH that is not corrected by ART. However, previous studies have not conducted a metabolic analysis comprehensive enough to determine the energetic pathways that are perturbed by HIV infection, and those which are corrected or not corrected by ART. Glucose can be processed through glycolysis, PPP, and TCA cycle for energy production. We found that serum glucose and G-6P levels were lower in pre-ART PWH and only partially normalized with ART. G-6P is further metabolized into various substrates by glycolysis to produce pyruvate or by is metabolized by an alternative hexose monophosphate shunt (also known as the PPP). PPP uses a series of enzyme catalyzed reactions to drive glucose utilization towards anabolic reactions that support bimolecular synthesis rather than ATP production. We found PPP products R-5P and E-4P were upregulated in the serum of pre-ART PWH and were not fully normalized by ART, suggesting that HIV infection upregulated the activity of PPP that was not corrected by ART. Based on the evidence that glucose-6-phosphate dehydrogenase (G6PDH) expression is increased following HIV infection in U937 macrophages [31], and in CD4+ T cells [32], it is possible that an upregulation of G6PDH is responsible for this particular bioenergetic adaptation. Indeed, G6PDH catalyzes the rate-limiting reaction that coverts G-6P in the PPP. HIV may upregulate PPP to promote the de novo synthesis of nucleotides, coenzyme A, and other biomolecules that are required to support infection [7,32]. PPP also plays an important role in maintaining viral latency in lymphoid and myeloid cells [31,32] by producing nicotinamide adenine dinucleotide (NADPH) that is required to fuel antioxidant pathways that contribute to the maintenance of HIV latency, and protect infected cells from oxidative stress-induced apoptosis [32]. These findings suggest that HIV infection shifts the cellular energetic preference to favor PPP over glycolysis to create a cellular environment that supports HIV replication and latency, while preventing infected cells from undergoing apoptosis. This preference for PPP was still apparent in PWH on ART with undetectable viral loads, suggesting that residual HIV replication is not responsible for this immunometabolic reprogramming.

### 5.2. TCA Cycle Impairment

Pyruvate is an end-product of glycolysis that can be fermented into lactate in the cytosol or can enter into mitochondria to form acetyl-CoA that fuels the TCA cycle via oxidative phosphorylation for energy production. Although pyruvate levels were not modified by HIV infection, lactate levels were higher in pre-ART PWH and were not completely normalized by ART. While the fermentation of pyruvate into lactate only produces two molecules of ATP per reaction, it is a rapid reaction that can produce considerable quantities of ATP in a relatively short time frame to meet the increased energy demand that occurs with HIV infection. ART did not fully restore glycolysis, lactate production, and PPP metabolism among post-ART PWH. Based on the correlations of these metabolite levels with CD4+ T-cell counts in both pre-ART and post-ART PWH, it is possible that the amount of bioenergetic impairment in PWH is partially dependent on the energetic demand required for immunometabolism. It is also possible that ART contributed to these bioenergetic impairments, as NRTI use has been associated with increased lactate levels in PWH [23,33,34]. In this cohort, a large number of PWH were treated with ART regimens containing lamivudine (3TC-NRTI), suggesting that the residual impairments in glycolysis may in part be attributable to NRTI use.

Mitochondrial TCA cycle intermediates not only serve as energetic substrates, but are also synthetic precursors of other products, including fatty acids, amino acids, and nucleotide bases [35]. In this study, pyruvate levels in pre-ART PWH were similar to HIV_SN_, but citrate and aconitate levels were decreased and α-KG levels increased. In the setting of HIV infection, citrate may be shuttled away from the TCA cycle (cataplerosis) for the synthesis of specific lipids and proteins that are required for immune cell differentiation and HIV-1 replication [33,34,36]. For example, there is evidence that lipid metabolism is altered in HIV-1-infected cells to support the establishment of membrane microdomains that are sites for HIV-1 assembly and budding [37,38]. Citrate derived acetyl-CoA can also be used to initiate the acylation of proteins that are required for cytokine production. For example, histone acylation is required for IFN-γ production, a critical regulatory factor of adaptive immunity that is increased during HIV infection [39,40]. To replenish TCA cycle under conditions of citrate metabolic reprogramming, other sources of interconnected metabolic pathways are evoked to support the generation of TCA cycle products. For example, glutaminolysis (anaplerosis) may occur in which amino acid de-amination reactions lead to the generation of alpha-keto acids (i.e., α-KG from glutamate). Recent studies demonstrate an upregulation of glutaminolysis in HIV [8] and in other viral infections [41], suggesting that the increased levels of α-KG in PWH might be due to the upregulation of glutaminolysis to support a compromised TCA cycle. These data suggest that HIV infection compromises the TCA cycle with a notable increase in the cataplerosis of citrate to support the biosynthesis of lipids and other macromolecules, with an associated increase in the anaplerosis of glutamine to replenish the TCA cycle in the form of α-KG. ART was unable to completely normalize the abnormalities in TCA cycle metabolism that occurred with HIV infection. In PWH who had undetectable viral loads, serum glutamate levels normalized while α-KG remained elevated, suggesting that the complete suppression of HIV replication further improved, but did not completely normalize, TCA cycle dysregulation.

### 5.3. Impairments in β-Oxidation

Dyslipidemia and lipodystrophy are prevalent in PWH, particularly in individuals on PIs [42,43]. Lipid metabolism is tightly controlled through multiple integrated biochemical pathways that regulate fat mobilization and utilization. In this study, we observed that serum LCSFFAs (carbon chains >10) were higher, and their corresponding FACs (C12-C18) were lower in pre-ART PWH compared with HIV_SN_. In contrast, the medium-chain fatty acid octanoate and its corresponding FAC (ocatnoyl carnitine) were lower in pre-ART PWH compared with HIV_SN_, and were not fully normalized by ART. In general, FACs are intermediate substrates in the oxidative catabolism of fatty acids. We found a decrease in the ratio of fatty acylcarnitine to their corresponding fatty acids C12-C18 (laurate, myristate, palmitate, and stearate) in pre-ART PWH compared with HIV_SN_, none of which were completely normalized by ART. We also observed that β-hydroxybutyrate, a ketogenic product formed via acetyl CoA that is derived from fatty acid β-oxidation, was lower in pre-ART PWH, and completely normalized by ART. These data suggest that fatty acid utilization through β-oxidation is impaired in pre-ART PWH and is not fully restored by ART. These findings are supported by previous reports showing decreased levels of acylcarnitines in PWH [44] and the ratio of acylcarnitine-to-FFA in post-ART PWH [45]. Since small- and medium-chain fatty acids enter into mitochondria via a carnitine-independent pathway, and LCSFFAs must conjugate with carnitine to facilitate their entry into mitochondria for oxidative metabolism [46], our findings suggest that only LCSFFA utilization is impaired in PWH and is accompanied by a compensatory overutilization of medium-chain fatty acids. We also observed lower circulating levels of free carnitine among pre-ART PWH, however they were not completely normalized by ART, suggesting the mechanism for this shift in fatty acid utilization may be due impairment in fatty acylcarnitine binding or mitochondrial dysfunction among PWH.

Although there is a sufficient carnitine pool in healthy individuals, PWH are at a greater risk of carnitine deficiency due to the malabsorption of nutrients, renal disease with subsequent higher carnitine elimination, increased metabolic demands that result from higher circulating fatty acid levels, and reduced endogenous biosynthesis [47]. Indeed, adults and children with HIV infection exhibit lower levels of total circulating carnitines [48,49,50]. In a study, the dietary supplementation of 2 g of L-carnitine for 8 months improved fatty acid oxidation, with reduced levels of circulating free fatty acids in PWH with lipodystrophy receiving ART [51]; however, more investigation is required to conclude these promising findings, whether carnitine supplementation may improve fatty acid oxidation metabolism associated with dyslipidemia among PWH. In this study, serum carnitine levels were completely normalized in PWH after ART in those with an undetectable viral load; in addition, we found that impairments of β-oxidation of LCSFFAs in PWH after ART were slightly improved in those with undetectable viral loads, but not fully normalized.

### 5.4. Amino Acid Catabolism

One additional potential mechanism for reduced carnitine levels is a reduction in two essential amino acids, lysine and methionine, which were decreased in pre-ART PWH. However, ART returned lysine and methionine to levels similar to HIV_SN_. Lysine and methionine are precursors for the de novo synthesis of carnitine [52]. Although the metabolism of amino acids primarily regulates anabolic reactions for protein synthesis, some amino acids, including branched-chain amino acids (BCAAs: leucine, isoleucine, and valine), glutaminolysis amino acids (glutamate and glutamine), and alanine, are mobilized under pathological conditions to serve as alternative energetic substrates [53]. We found that lysine and methionine were significantly reduced, while leucine, isoleucine, valine, glutamine, glutamate, and alanine were significantly elevated in pre-ART PWH compared to HIV_SN_. ART resulted in a normalization of all amino acids, with the exception of glutamate and alanine, to levels observed in HIV_SN_. In pre-ART PWH, levels of serum α-KG and α-ketoisovalerate (α-KIV, a deaminated product of valine) along with glutamine, glutamate, and BCAAs were increased, suggesting that the elevated amino acids in serum may be due to increased proteolysis associated with muscle wasting in PWH. This assumption is supported by elevated levels of serum hydroxyproline, a marker of proteolysis [54,55] that was elevated in pre-ART PWH compared with HIV_SN_.

Although the biological mechanism for muscle wasting in PWH is not entirely understood, a glucose-alanine shuttle (also known as the Cahill cycle) is known to operate under conditions of bioenergetic stress. In this system, alanine produced in the muscle from protein breakdown is transported to the liver for gluconeogenesis, and the glucose generated is transported back to the muscle to fulfill muscle energetic demand [56,57,58]. Other substrates, including valine, glutamine, glutamate, and α-KG, which we observed to be increased in PWH, also contribute to the glucose-alanine cycle [56,57]. These results suggest that a bioenergetic surge associated with HIV-1 infection may lead to protein degradation in skeletal muscles to mobilize amino acids and their catabolic products into circulation. Following the initiation of ART, levels of serum amino acids largely normalized among post-ART PWH with the exception of glutamate and alanine, suggesting that gluconeogenesis and the glucose–alanine shuttle may remain elevated in PWH receiving ART, albeit to a far lesser degree. Unfortunately, there are no detailed studies pertaining to BCAAs oxidative metabolism in HIV pathology, and further studies are necessary to evaluate this premise. The serum levels of glutamate were completely normalized in PWH with undetectable viral loads, but alanine remained elevated (partially improved) among post-ART PWH.

### 5.5. Association of Serum Bioenergetics with Clinical Markers

The correlational analysis suggests that serum levels of glycolysis substrates, PPP, and TCA cycle products are largely influenced by CD4+ count, while fatty acid and amino acid catabolic products are influenced by serum viral loads among pre- and post-ART PWH. These findings suggest that serum levels of energy metabolites reflect disease status and immunometabolism in PWH.

### 5.6. Implications of Bioenergetic Shifts in PWH

Serum metabolic fuels, including primary and alternative energetic substrates, such as glucose, fatty acids, and amino acids, and their corresponding secondary metabolites, which include lactate, PPP products, TCA cycle intermediates, fatty acylcarnitines, ketone bodies, and α-keto acids, not only reflect immunometabolic status, but also may serve as prognostic markers predicting the course of a disease. Abnormalities in immunometabolism are associated with a metabolic syndrome that is common in PWH [45,59,60,61,62,63]. Chronic alterations in immunometabolism may contribute to a variety of comorbid conditions, including insulin resistance [49,64,65,66], obesity [64,67], cardiovascular disease [65,66,68], type-2 diabetes [66,69], lipodystrophy [60,70], liver complications (hepatic insulin resistance, viral hepatitis, and steatohepatitis) [71,72], cancer [73,74], and central nervous system complications [75,76,77].

### 5.7. Strengths and Limitations of the Study

To our knowledge, this is the first study to report the bioenergetic adaptations associated with immunometabolism in PWH before and after ART. The participants were equally distributed by sex, largely drug free, and with limited alcohol use. This unique study population allowed us to determine the effects of HIV infection and the impact of ART on immunometabolism with minimal confounders. The limitations of the study include a lack of follow-up samples from the HIV_SN_ group and differences in ART initiation among PWH based on the changes in Ugandan guidelines for ART initiation that rose from a CD4 cell count of 200 to 500 CD4/mL. Although we determined if the associations were modified by removing PWH that did not achieve full viral suppression, we did not separately evaluate those with the baseline CD4 cell count <200 (advanced immunosuppression) from those with CD4 baselines between 350–500 (moderate immunosuppression) due to insufficient power.

## 6. Conclusions

HIV-1 infection was associated with dysregulations in multiple bioenergetic pathways associated with immunometabolism, including increased glucose utilization, the upregulation of PPP, increased lactate production, impairments in fatty acid β-oxidation, and the upregulation of amino acid oxidative catabolism. ART only partially restored these impairments in bioenergetics consistent with chronic disruptions in immunometabolism, despite viral suppression. Based on the known associations of these metabolic abnormalities with a number of pathological conditions, it is likely that the increased rate of co-morbid conditions in PWH involves a sustained metabolic and immune dysregulation that is not corrected by ART. Metabolic interventions focusing on PPP and/or de novo lipogenesis may improve clinical outcomes among PWH receiving ART in part by regulating immune function. However, further research is necessary to validate these findings and to understand the roles for dysregulated immunometabolism in the development of non-AIDS comorbid conditions in PWH.

## Figures and Tables

**Table 1:** The demographic and clinical characteristics of the study participants.

Characteristics	HIV_SN_	Pre-ART PWH	*p*-Value	Pre-ART PWH	Post-ART PWH	*p*-Value	HIV_SN_	post-ART HIV	*p*-Value
**Gender**									
**Female (F)**	143	142		142	142		143	142	
**Male (M)**	157	136		136	136		157	136	
**Age (years) [mean (SD)]**	35(8.1)	35.7(8.8)	0.89	35.7(8.8)	37.6(8.8)	**<0.01**	35(8.1)	37.6(8.8)	**0.022**
**Average BMI [mean (SD)]**	23.1(3.8)	22.3(3.3)	**0.03**	22.3(3.3)	22.9(3.2)	0.90	23.1(3.8)	22.9(3.2)	0.076
**Obese [*n* (%)]**	17(5.7)	9(3.2)		9(3.2)	12(4.3)		17(5.7)	12(4.3)	
**Blood pressure**									
**Systolic (mmHg)**	120(14)	115.3(41.5)	**<0.01**	115.3(41.5)	112.8(12.7)	**<0.01**	120(14)	112.8(12.7)	**<0.01**
**Diastolic (mmHg)**	71(10)	69.9(25.1)	0.47	69.9(25.1)	70.7(25.4)	0.98	71(10)	70.7(25.4)	0.604
**Hypertension [*n* (%)]**	50(16.7)	25(9.0)		25(9.0)	24(8.6)		50(16.7)	24(8.6)	
**HDL (g/L)**	1.2(0.4)	0.8(0.4)	**<0.01**	0.8(0.4)	1.2(0.4)	0.05	1.2(0.4)	1.2(0.4)	<0.01
**LDL (g/L)**	2.2(0.9)	2(0.8)	**0.02**	2(0.8)	2.3(0.8)	0.15	2.2(0.9)	2.3(0.8)	<0.01
**Triglycerides (g/L)**	1.1(0.5)	1.2(0.5)	0.38	1.2(0.5)	1.2(0.7)	0.30	1.1(0.5)	1.2(0.7)	0.934
**Cholesterol (g/L)**	4.1(1.2)	3.6(1.0)	**<0.01**	3.6(1.0)	4.4(2.3)	0.17	4.1(1.2)	4.4(2.3)	<0.01
**Dyslipidemia [*n* (%)]**	123(41)	234(78)		234(78)	110(39.6)		123(41)	110(39.6)	
**Fasting glucose (g/L)**	91.0(24.1)	87.4(13.3)	**0.04**	87.4(13.3)	89.5(16)	0.97	91.0(24.1)	89.5(16)	**<0.05**
**Diabetes [*n* (%)]**	6(2)	5(1.8)		5(1.8)	3(1.1)		6(2)	3(1.1)	
**CD4 T-cell count (advanced immunosuppression)** **(Median (IQR))**	NA	111(112)	NA	111(112)	279(167.5)	**<0.01**	NA	279(167.5)	NA
**CD4 T-cell count (moderate immunosuppression)** **(Median (IQR))**	NA	418(71)	NA	418(71)	512.5(214.7)	**<0.01**	NA	512.5(214.7)	NA
**log_serum_vl_copies (copies/mL) (Median (IQR))**	NA	4.58(1.2)	NA	4.58(1.2)	0.00(0.0)	**<0.01**	NA	0.00(0.00)	NA
**Therapy regimen (months)**	NA	NA		NA	720(676–882)		NA	720(676–882)	
**TDF/3TC/EFV**	NA	NA		NA	256		NA	256	
**TDF/3TC/NVP**	NA	NA		NA	10		NA	10	
**AZT/3TC/EFV**	NA	NA		NA	3		NA	3	
**AZT/3TC/NVP**	NA	NA		NA	9		NA	9	
**Liver parameters**									
**ALT (IU/mL)**	0.98(0.3)	0.97(0.3)	0.73	0.97(0.3)	1.3(0.3)	**<0.01**	0.98(0.3)	1.3(0.3)	**<0.01**
**AST (IU/mL)**	1.3(0.2)	1.4(0.2)	**<0.01**	1.4(0.2)	1.5(0.2)	**<0.01**	1.3(0.2)	1.5(0.2)	**<0.01**

## Data Availability

Anonymized data not published within this article will be made available at the request from any qualified investigator.

## Supplementary Materials

The following supporting information can be downloaded at: https://www.mdpi.com/article/10.3390/v14061313/s1, Figure S1: Associations between serum metabolites, demographics and liver function in HIV_SN_. Heatmap visualizations of the correlations between bioenergetics (A) glucose metabolism, (B) Fatty acid oxidation, (C) amino acid metabolism and age, gender, biomarkers of liver function (ALT and AST) among HIV_SN_. Numbers in the heatmap cells are Pearson’s correlation coefficient R-values. Blue represents positive correlations, and red represents negative correlations. Figure S2: Serum levels of bioenergetic substrates implicated in immunometabolism. Serum levels of (A) aconitate, (B) succinate, (C) glutamate, (D) carnitine, (E) deaconate, and (F) alanine in PWH before and after ART with undetectable viral load compared with HIV_SN_. Scatterplots show the mean, and significance notations of adjusted *p* values (95% CI) at * = *p* < 0.05, ** = *p* < 0.01, *** = *p* < 0.001, and **** = *p* < 0.0001. For multiple group comparisons Brown-Forsythe and Welch ANOVA tests were performed in combination with Games-Howell multiple comparisons correction for >50 sample size with a 95% confidence interval. Figure S3: Associations between serum metabolites, demographics and clinical markers in post-ART PWH with undetectable viral loads. Heatmap visualizations of the correlations between bioenergetics (A) glucose metabolism, (B) Fatty acid oxidation, (C) amino acid metabolism and age, gender, biomarkers of liver function (ALT and AST), and biomarkers of HIV disease status (CD4+ T cell count and HIV viral load) among post-ART PWH with undetectable viral loads. Numbers in the heatmap cells are Pearson’s correlation coefficient R-values. Blue represents positive correlations, and red represents negative correlations. The levels of Glucose (r = 0.47), G-6P (r = 0.60), citrate (r = 0.56), aconitate (r = 0.58), and succinate (r = 0.62), lysine (r = 0.51), isoleucine (r = 0.52) were positively correlated, while lactate levels (r = −0.6) negatively correlated with CD4+ T cell counts among post-ART PWH with undetectable viral loads. The raw mass spectral data of serum metabolites, demographics, and clinical data are provided in Excel sheet.
