# Supplementary material for: Immunometabolic Reprogramming in Response to HIV Infection Is Not Fully Normalized by Suppressive Antiretroviral Therapy
Source: Viruses. 2022 Jun 15;14(6):1313. doi: 10.3390/v14061313 (PMC9228482; doi:10.3390/v14061313)
Supplement: Supplementary file 1 [file viruses-14-01313-s001.zip › Immunometabolism Supplementary Figures.pptx]

## Slide 1
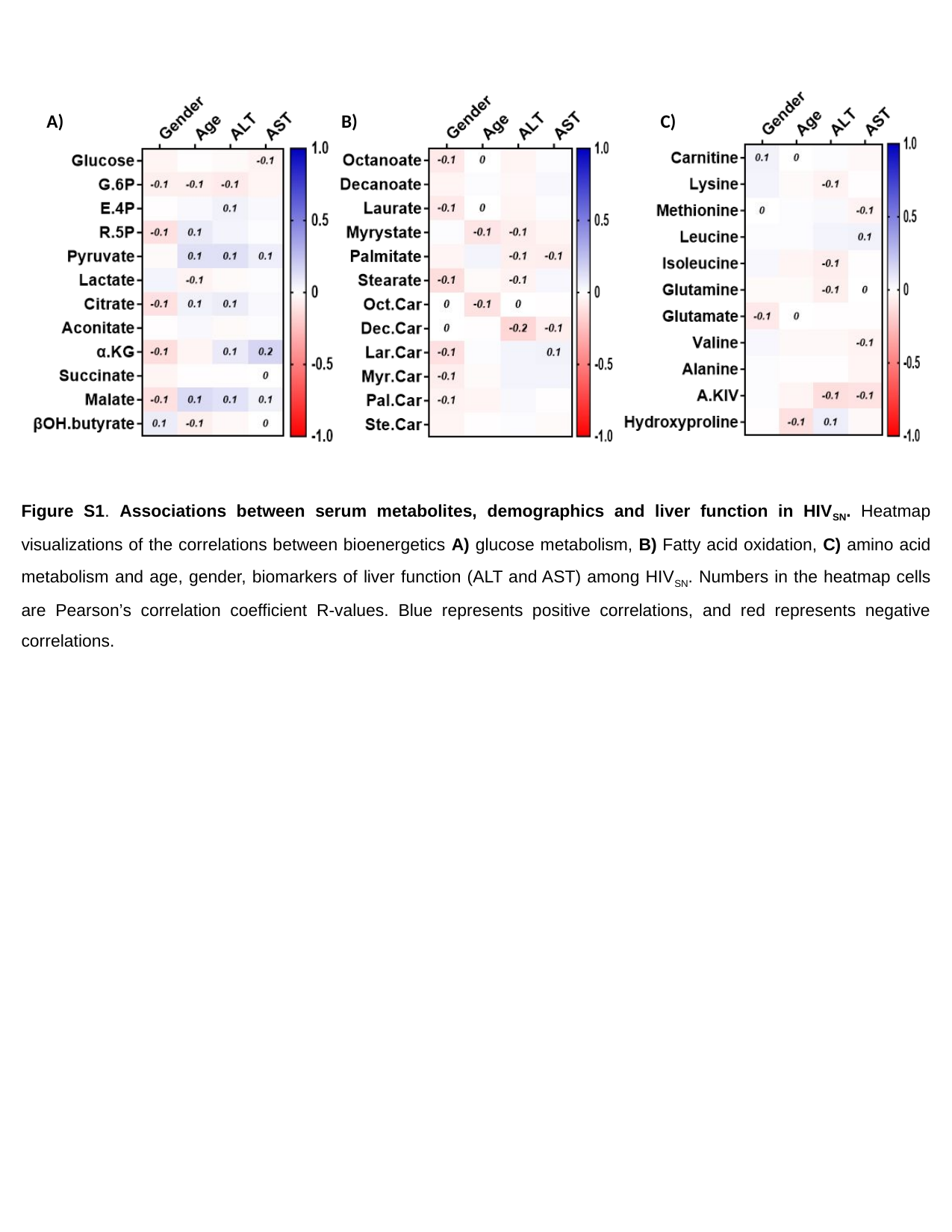

A)
B)
C)
Figure S1. Associations between serum metabolites, demographics and liver function in HIVSN. Heatmap visualizations of the correlations between bioenergetics A) glucose metabolism, B) Fatty acid oxidation, C) amino acid metabolism and age, gender, biomarkers of liver function (ALT and AST) among HIVSN. Numbers in the heatmap cells are Pearson’s correlation coefficient R-values. Blue represents positive correlations, and red represents negative correlations.

## Slide 2
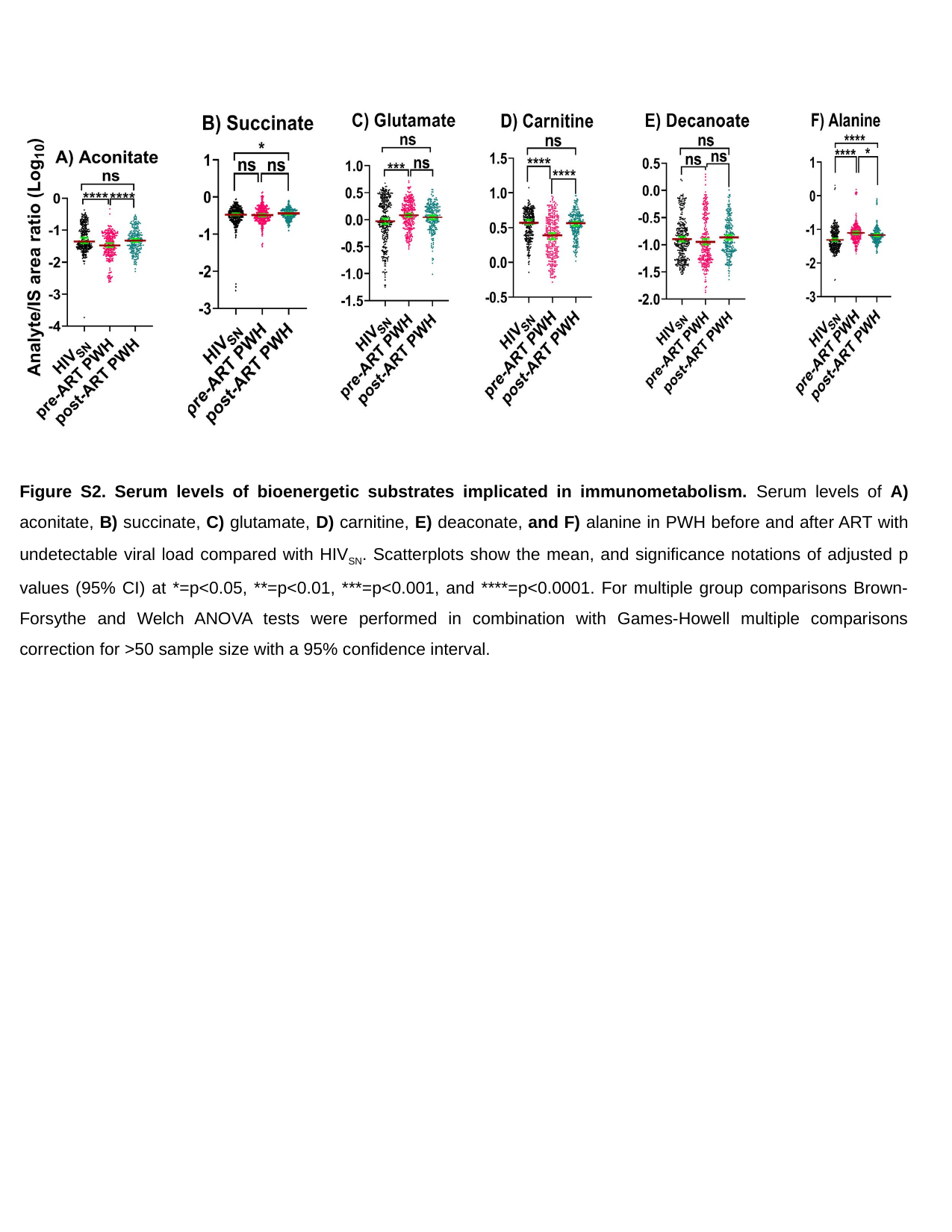

Figure S2. Serum levels of bioenergetic substrates implicated in immunometabolism. Serum levels of A) aconitate, B) succinate, C) glutamate, D) carnitine, E) deaconate, and F) alanine in PWH before and after ART with undetectable viral load compared with HIVSN. Scatterplots show the mean, and significance notations of adjusted p values (95% CI) at *=p<0.05, **=p<0.01, ***=p<0.001, and ****=p<0.0001. For multiple group comparisons Brown-Forsythe and Welch ANOVA tests were performed in combination with Games-Howell multiple comparisons correction for >50 sample size with a 95% confidence interval.

## Slide 3
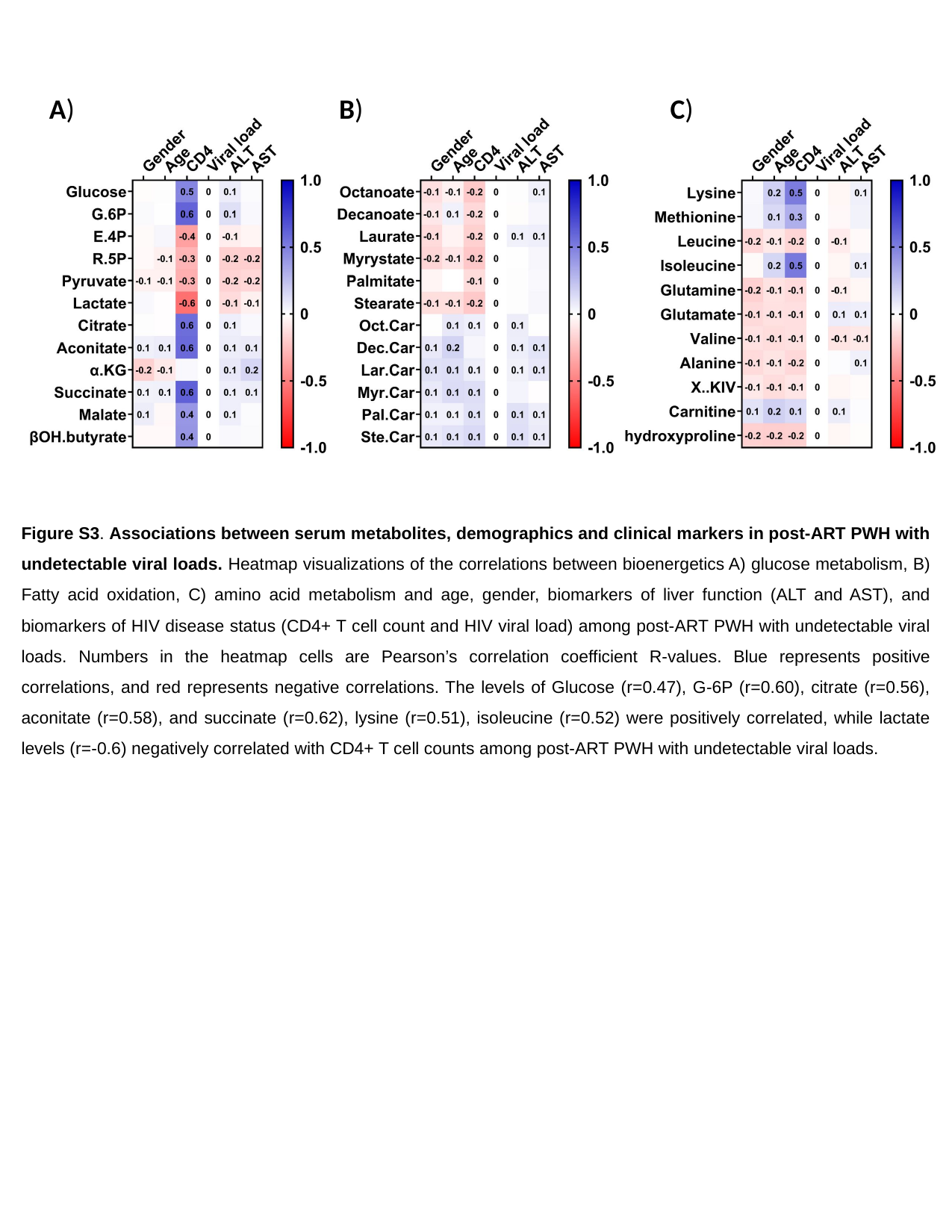

B)
C)
A)
Figure S3. Associations between serum metabolites, demographics and clinical markers in post-ART PWH with undetectable viral loads. Heatmap visualizations of the correlations between bioenergetics A) glucose metabolism, B) Fatty acid oxidation, C) amino acid metabolism and age, gender, biomarkers of liver function (ALT and AST), and biomarkers of HIV disease status (CD4+ T cell count and HIV viral load) among post-ART PWH with undetectable viral loads. Numbers in the heatmap cells are Pearson’s correlation coefficient R-values. Blue represents positive correlations, and red represents negative correlations. The levels of Glucose (r=0.47), G-6P (r=0.60), citrate (r=0.56), aconitate (r=0.58), and succinate (r=0.62), lysine (r=0.51), isoleucine (r=0.52) were positively correlated, while lactate levels (r=-0.6) negatively correlated with CD4+ T cell counts among post-ART PWH with undetectable viral loads.
